# Predicting Drug-Target Interactions via Within-Score and Between-Score

**DOI:** 10.1155/2015/350983

**Published:** 2015-10-12

**Authors:** Jian-Yu Shi, Zun Liu, Hui Yu, Yong-Jun Li

**Affiliations:** ^1^School of Life Sciences, Northwestern Polytechnical University, Xi'an, Shaanxi 710072, China; ^2^School of Computer Science, Northwestern Polytechnical University, Xi'an, Shaanxi 710072, China

## Abstract

Network inference and local classification models have been shown to be useful in predicting newly potential drug-target interactions (DTIs) for assisting in drug discovery or drug repositioning. The idea is to represent drugs, targets, and their interactions as a bipartite network or an adjacent matrix. However, existing methods have not yet addressed appropriately several issues, such as the powerless inference in the case of isolated subnetworks, the biased classifiers derived from insufficient positive samples, the need of training a number of local classifiers, and the unavailable relationship between known DTIs and unapproved drug-target pairs (DTPs). Designing more effective approaches to address those issues is always desirable. In this paper, after presenting better drug similarities and target similarities, we characterize each DTP as a feature vector of within-scores and between-scores so as to hold the following superiorities: (1) a uniform vector of all types of DTPs, (2) only one global classifier with less bias benefiting from adequate positive samples, and (3) more importantly, the visualized relationship between known DTIs and unapproved DTPs. The effectiveness of our approach is finally demonstrated via comparing with other popular methods under cross validation and predicting potential interactions for DTPs under the validation in existing databases.

## 1. Introduction

Since experimental determination of compound-protein interactions or potential drug-target interactions remains very challenging (e.g., requiring a huge amount of money and taking a very long period) [1], there is a need to develop computational methods to assist those experiments. Nowadays, the number of available drug-target interactions (DTIs) in public database, including KEGG [2], PubChem [3], DrugBank [4], and ChEMBL [5], is increasing which brings out two observations. The first one is that one drug can interact with one or more proteins. Another is symmetrically the fact that one protein can be targeted by one or more drugs. These two observations led to the formation of DTI network [6] and made it possible to utilize DTIs (approved drug-target pairs) to predict potential interactions among unapproved drug-target pairs (DTPs). The task to validate those predicted potential interactions is called drug repositioning or drug repurposing [7].

In terms of DTI network, predicting newly potential DTI is equivalent to predicting new edges in the network. Researchers developed network-based inference model (NBI) to deduce the potential interactions among unapproved DTPs in given DTI networks and further confirmed them from* in vitro* assays [7]. However, NBI cannot run the prediction for any DTP between which no reachable path (a set of consecutively connected edges) in network is available. In fact, a DTI network usually contains several isolated subnetworks. A difficult case for NBI is, for example, to predict the interaction between the drug in one subnetwork and the target in another. Besides, predicting interactions for a drug node d, the resulting targets usually bias to the target nodes of more degrees or the target nodes near to drug d.

With a different idea of regarding similarity matrices of drugs and targets as kernel matrices, kernel-based techniques of classification, such as bipartite local model (BLM) [8–10], are also popularly applied to DTI prediction. As a local classification model, for each target, BLM assigns known DTIs and unapproved DTPs between drugs and the concerned target as positive and negative samples, respectively. Then a kernel-based classifier is built on drug similarity matrices and applied to assign confidence scores to unlabeled samples (concerned unapproved DTPs). Similarly, for each drug, another kernel-based classifier can be also built. For each drug-target pair, we need to build two classifiers of which the output scores further are aggregated as the final score [8, 9]. BLM, however, generates the biased prediction in the case of few positive samples (known DTIs). Also it cannot predict the interaction between a new drug (without linking to any known target) and a new target (without linking to any known drug) because no positive samples are available to train its classifier model. BLM-NII, an extension of BLM, recently developed a weighted strategy and integrated it into BLM to tackle the case of no positive sample available [10]. However, the biased prediction still remains when few positive samples are available. More importantly, since drug-target pairs are separately put into different classifier spaces, neither BLM nor BLM-NII is able to investigate the relationship between them. Such relationship is helpful for further predicting the potential interactions in both drug discovery and drug repositioning.

To summarize, three issues in existing predictive models are not yet solved. (1) Predicting interactions between drugs and targets occurring in isolated subnetworks of DTI network is difficult. (2) Inadequate positive samples usually cause biased local classifiers and local classification approach requires a number of classifiers. (3) The global relationship between approved DTIs and unapproved DTPs cannot be investigated in a consistent space.

Except for the predictive model, similarity measuring is another crucial factor in DTI prediction because similar drugs tend to interact with similar targets [11]. To capture pairwise similarities between drugs or targets in a better way, a topological similarity based on DTI network was proposed, such as Gaussian interaction profile (GIP) [9] and was linearly integrated into chemical structure-based similarity between drugs or protein sequence-based similarity between targets under the framework of BLM. Nevertheless, simple linear combination may not work optimally because the topological similarity is always related to the drug/target node degrees, which follow the power-law distribution [12]. In addition, for any two drugs/targets, GIP only considers the targets/drugs not interacting with them but has no consideration of the targets/drugs shared by them. So GIP may lose some information derived from those common targets/drugs between drugs/targets. Besides, since all possible values of the topological similarity proposed in GIP falls into (0, 1], GIP is an incomplete similarity metric which may not adequately characterize the dissimilarity between those very different drugs/targets.

In this paper, we believe that the difference between the similarities of drugs/targets sharing targets/drugs and the similarities of drugs/target sharing no target/drug in DTI network should be statistically significant. To address abovementioned issues, we first characterized each drug-target pair from the views of both drugs and targets, respectively. Under the publicly acceptable assumption that similar drugs tend to target similar protein receptors [11], two within-scores were presented to capture the similarities between drugs/targets sharing common targets/drugs. Based on our observation that similar drugs, in part, do not tend to target dissimilar proteins, two between-scores were also presented to capture the similarities between drugs/targets share no targets/drugs.

Subsequently, we represented each drug-target pair as a feature vector which uniformly consists of four scores, regardless of the available path between drugs and targets. Each drug-target pair was labeled as positive or negative sample, depending on whether it is an approved DTI or an unapproved DTP. The use of all DTIs can guarantee that enough positive samples can be used to train the only one global classifier. After performing principal component analysis on feature vectors, we generated a drug-target pair space which provides a visualized way to investigate the relationship between known DTIs and unapproved DTPs.

In addition, to obtain a better combination between topological similarity and chemical/sequence similarity, we proposed an adaptive combination rule instead of the former linear combination and introduced a complete metric of topological similarity of drugs/targets by considering both the targets/drugs shared by two drugs/targets and the targets/drugs interacting with none of them.

Finally, based on four benchmark datasets, we demonstrated the effectiveness of our approach, by comparing with NBI, BLM, and BLM's extensions in cross validation and predicting potential interactions in unapproved DTPs under checking in existing databases.

## 2. Materials and Method

### 2.1. Datasets

In this paper, the adopted datasets, involving targets of ENZYME, ION CHANNEL, GPCR, and NUCLEAR RECEPTOR, were originally from [13] and further used in subsequent works [8–10]. All of drug-target interactions in the original datasets were collected from KEGG database. In short, we denote the four DTI datasets as EN, IC, GPCR, and NR, respectively. The brief information of four datasets is listed in Table 1. Notably, NR (the sparest DTI network in the given datasets) contains the most proportions of isolated subnetworks and is the most difficult case to predict the potential DTI [10] because it has most the proportion of unreachable paths between drugs and between targets. More details can be found in the original work [13].

### 2.2. Drug Similarity and Target Similarity

The metrics of drug similarity and target similarity popularly adopted in former methods are chemical structure-based similarity and protein sequence-based similarity, respectively [8–10]. By representing a chemical structure as a graph, the chemical structure similarity between two drugs is defined as *S*_*d*_^chem^(*d*_*u*_, *d*_*v*_) = |*d*_*u*_∩*d*_*v*_|/|*d*_*u*_ ∪ *d*_*v*_|, where |·| denotes the number of nodes in graph, *d*_*u*_∩*d*_*v*_ is the maximal common subgraph between *d*_*u*_ and *d*_*v*_, and *d*_*u*_ ∪ *d*_*v*_ is their union [14]. The protein sequence similarity between two targets is calculated by sequence alignment and is defined as $S_{t}^{\text{seq}}(t_{u},t_{v})=\text{align}(t_{u},t_{v})/\sqrt{\text{align}(t_{u},t_{u})\text{align}(t_{v},t_{v})}$, where align(*t*_*u*_, *t*_*v*_) is the Smith-Waterman alignment score [15] between *t*_*u*_ and *t*_*v*_.

In order to capture the real similarity between drugs/targets sharing common targets/drugs in a better way, former methods tried to propose new similarities and integrate them into abovementioned similarities. Under the framework of BLM, Gaussian interaction profile (GIP) was introduced to measure topological similarity between drugs/targets by considering DTI matrix as the adjacent matrix of DTI network [9]. However, for any two drugs/targets, GIP only considers the targets/drugs not interacting with them so that it may lose some information derived from their common targets/drugs. In addition, GIP is not a mathematically complete similarity since its similarity values fall into (0, 1]. So it may not be enough to characterize the dissimilarity between very different drugs/targets. Therefore, we applied a complete metric to measure the similarities between nodes of both drugs and targets, respectively, according to the DTI network. The topological similarity, named matching index (MI) [16], between drugs and the topological similarity between targets are defined as follows:

(1) $$\begin{array}{c} S_{d}^{\text{topo}}\left(d_{i},d_{j}\right)=\frac{T-\left|d_{i}\right|-\left|d_{j}\right|+2\left|d_{i}\cap d_{j}\right|}{T},\\ S_{t}^{\text{topo}}\left(t_{p},t_{q}\right)=\frac{D-\left|t_{p}\right|-\left|t_{q}\right|+2\left|t_{p}\cap t_{q}\right|}{D}, \end{array}$$

where |·| denotes the degree of nodes and |*x*∩*y*| is the number of sharing neighbors of two nodes. For drugs, *S*_*d*_^topo^(*d*_*i*_, *d*_*j*_) considers the proportion of their shared target nodes as well as target nodes not interacting with them. For targets, *S*_*t*_^topo^(*t*_*p*_, *t*_*q*_) holds the similar consideration. Moreover, all possible values of MI fall into [0,1].

In former work [9], the final similarities of drug and target are usually generated by linearly combining *S*_*d*_^topo^ and *S*_*t*_^topo^ with *S*_*d*_^chem^ and *S*_*d*_^topo^, respectively. Nevertheless, such linear combination may not work optimally because the topological similarity is always related to the node degrees which follows the power-law distribution [12].

We observed that the topological similarity always works better when those drugs link to a target node of small degree; in contrast, chemical similarity always works better when those drugs link to a target node of large degree, respectively. Consequently, we designed an adaptive combination rule to expectedly achieve better prediction for MI. For target *t*_*p*_ linking to *g*_*t*_*p*__ drugs, the similarity between *d*_*i*_ and *d*_*j*_ among *g*_*t*_*p*__ drugs is defined as follows:

(2) Sddi,dj  =Sdchemdi,djgtp≥utmax⁡Sdchemdi,dj,Sdtopodi,djlt<gtp<utSdtopodi,djgtp≤ltut=0.5∗max⁡gtp, lt=∑p=1TgtpT, p=1,…,T.

The similarity between targets *t*_*p*_ and *t*_*q*_ can be defined in the similar way.

### 2.3. Within-Score and Between-Score of a Drug-Target Pair

A publicly acceptable assumption is that similar drugs tend to target similar protein receptors [11]. Based on this assumption, by considering the similarities between drugs/targets sharing common targets/drugs, we shall present two within-scores to capture them. Based on our additional observation that similar drugs, in part, do not tend to target dissimilar proteins, we shall also propose two between-scores to capture the similarities between drugs sharing no target and the similarities between targets sharing no drug respectively. The calculation of within-scores and between-scores is depicted in the following paragraphs.

Given *D* drugs and *T* targets, and their known interactions, our task is to predict potential but unapproved interactions between drugs and targets. All drug-target pairs are usually organized as an interaction matrix *A*_*D*×*T*_, in which *a*_*ij*_ = 1 when there is a known interaction between drug *d*_*i*_ and target *t*_*j*_, and *a*_*ij*_ = 0 otherwise.

For drug *d*_*i*_ interacting with *T*_*i*_ targets, *t*_*p*_^*i*^ and $t_{q}^{\tilde{i}}$ denote the target interacting and not interacting with *d*_*i*_, respectively. In order to characterize the potential interaction *P*(*t*_*x*_, *d*_*i*_) between drug *d*_*i*_ and a queried target *t*_*x*_, we define within-score *C*_*t*_^*w*^(*t*_*x*_, *d*_*i*_) and between-score *C*_*t*_^*b*^(*t*_*x*_, *d*_*i*_) from drug view as follows:

(3) $$\begin{array}{c} C_{t}^{w}\left(t_{x},d_{i}\right)=\max\left(\left\{S_{t}\left(t_{x},t_{p}^{i}\right)\right\}\right),\;p=1,2,\ldots ,T_{i},\\ C_{t}^{b}\left(t_{x},d_{i}\right)=\max\left(\left\{S_{t}\left(t_{x},t_{q}^{\tilde{i}}\right)\right\}\right),\;q=1,2,\ldots ,T-T_{i}, \end{array}$$

where *S*_*t*_(*t*_*x*_, *t*_*p*_^*i*^) is the similarity between *t*_*x*_ and *t*_*p*_^*i*^ and $S_{t}(t_{x},t_{q}^{\tilde{i}})$ is the similarity between *t*_*x*_ and $t_{q}^{\tilde{i}}$. Then, the drug-view feature of *P*(*t*_*x*_, *d*_*i*_) is defined as *f*(*t*_*x*_, *d*_*i*_) = [*C*_*t*_^*w*^(*t*_*x*_, *d*_*i*_), *C*_*t*_^*b*^(*t*_*x*_, *d*_*i*_)].

For target *t*_*j*_ interacting with *D*_*j*_ drugs, *d*_*u*_^*j*^ is the drug interacting with it and $d_{v}^{\tilde{j}}$ is the drug not interacting with it. Symmetrically, from target view, we define within-score *C*_*d*_^*w*^(*d*_*y*_, *t*_*j*_) and between-score *C*_*d*_^*b*^(*d*_*y*_, *t*_*j*_) as follows:

(4) $$\begin{array}{c} C_{d}^{w}\left(d_{y},t_{j}\right)=\max\left(\left\{S_{d}\left(d_{y},d_{u}^{j}\right)\right\}\right),\;u=1,2,\ldots ,D_{j},\\ C_{d}^{b}\left(d_{y},t_{j}\right)=\max\left(\left\{S_{d}\left(d_{y},d_{v}^{\tilde{j}}\right)\right\}\right),\;v=1,2,\ldots ,D-D_{j}, \end{array}$$

where *S*_*d*_(*d*_*y*_, *d*_*u*_^*j*^) is the similarity between *d*_*y*_ and *d*_*u*_^*j*^ and $S_{d}(d_{y},d_{v}^{\tilde{j}})$ the similarity between *d*_*y*_ and $d_{v}^{\tilde{j}}$. Again, the target-view feature of the potential interaction *P*(*d*_*y*_, *t*_*j*_) is defined as *g*(*d*_*y*_, *t*_*j*_) = [*C*_*d*_^*w*^(*d*_*y*_, *t*_*j*_), *C*_*d*_^*b*^(*d*_*y*_, *t*_*j*_)]. Consequently, for the pair (*d*_*y*_, *t*_*x*_), we can obtain a combined feature vector:

(5) Fdy,tx  =[ftx,dy,gdy,tx]  =Ctwtx,dy,Ctbtx,dy,Cdwdy,tx,Cdbdy,tx.

### 2.4. Types of Interactions

**Figure 1:**
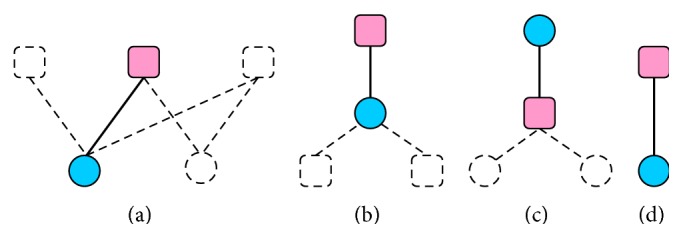
Topological motifs in drug-target network. (a) Multiple, (b) drug-centered, (c) target-centered, and (d) single pairs. Drugs and targets are denoted by circle nodes and rounded squares nodes, respectively. The pairs between concerned drugs (blue) and concerned targets (pink) are denoted by thick lines. The interactions between concerned nodes (filled by colors) and other nodes (hollow) are represented by dotted lines.

Totally, we group all interactions into four types according to DTI network (Figure 1): multiple, drug-centered, target-centered, and single interacting motifs. The summary of their counts in four adopted datasets can be found in Table S1 in Supplementary Material available online at http://dx.doi.org/10.1155/2015/350983.

Either the target or the drug of a multiple interaction has >1 links to drugs or targets, respectively. The target of a drug-centered interaction has only one link to the drug interacting with >1 targets. The drug of a target-centered interaction has only one link to the target interacting with >1 drugs. Both the target and the drug of a single interaction only link to each other. A single interaction is usually newly approved [6]. The drug-target pairs in multiple motif are just shown in formula 5 in previous section. The drug-target pairs involving in drug-centered, target-centered, and single motifs are the special cases of multiple motif and are shown as follows:

(6) $$\begin{array}{c} F_{d}\left(d_{y},t_{x}\right)=\left[C_{t}^{w}\left(t_{x},d_{y}\right),C_{t}^{b}\left(t_{x},d_{y}\right),\text{null},C_{d}^{b}\left(d_{y},t_{x}\right)\right],\\ F_{t}\left(d_{y},t_{x}\right)=\left[\text{null},C_{t}^{b}\left(t_{x},d_{y}\right),C_{d}^{w}\left(d_{y},t_{x}\right),C_{d}^{b}\left(d_{y},t_{x}\right)\right],\\ F_{s}\left(d_{y},t_{x}\right)=\left[\text{null},C_{t}^{b}\left(t_{x},d_{y}\right),\text{null},C_{d}^{b}\left(d_{y},t_{x}\right)\right], \end{array}$$

where null means that the score cannot be calculated directly. We adopted a bottom-line strategy to cope with the null cases by assigning ones to null entries.

With the representation of feature vector, we can map all drug-target pairs, including the pairs between new drugs and new targets, into the same space regardless of whether the drug and the target are in the same subnetwork or not.

### 2.5. Drug-Target Pair Space

**Figure 2:**
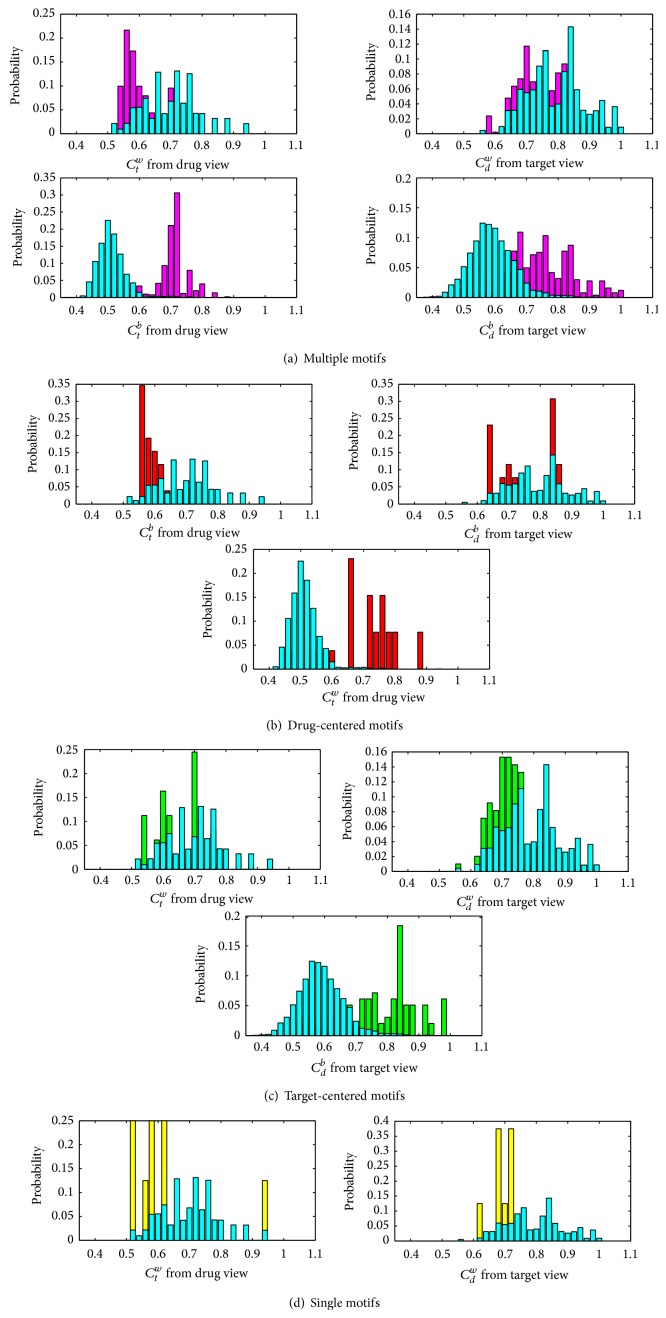
The distributions between known interactions (four types of motifs) and unapproved drug-target pairs. All histograms were generated by sorting scores into specific bins from 0.35 to 1.1. The *x*-axis in each histogram represents the bins with the intervals of 0.02. The *y*-axis denotes their heights which are the normalized counts (probabilities) of scores in corresponding bins. The histograms of multiple, drug-centered, target-centered, and single motifs are shown with purple, red, green, and yellow in (a), (b), (c), and (d), respectively. All histograms of unapproved drug-target pairs are rendered with cyan in all subfigures. The color of overlapping parts of two histograms in each subfigure is just the sum of their individual colors.

To check whether or not known interactions and unapproved pairs can be classified well in certain dimensions, we made the distributions of *C*_*t*_^*w*^, *C*_*d*_^*w*^, *C*_*t*_^*b*^, and *C*_*d*_^*b*^ scores in feature vectors by histograms for four types of DTIs. As an illustration, the score distributions of four motifs of GPCR dataset [13] are shown in Figure 2. The distributions of all datasets can be found in Figures S1, S2, S3, and S4.

Known DTIs and unapproved DTPs show separations in terms of distributions of four scores. That is to say, they can be classified in certain dimensions (scores). In detail, (1) for multiple motifs (Figure 2(a)), known interactions (purple) and unapproved DTPs (cyan) can be separated significantly by *C*_*t*_^*w*^, moderately separated by either *C*_*t*_^*b*^ or *C*_*d*_^*w*^, and almost mixed together in terms of *C*_*d*_^*b*^. (2) For drug-centered motifs whose *C*_*d*_^*w*^ is unavailable (Figure 2(b)), *C*_*t*_^*w*^, *C*_*t*_^*b*^, and *C*_*d*_^*b*^ show the best, the moderate, and the worst separations, respectively. (3) Likewise, for target-centered motifs whose *C*_*t*_^*w*^ is unavailable (Figure 2(c)), *C*_*d*_^*w*^ shows the best separation while neither *C*_*d*_^*b*^ nor *C*_*t*_^*w*^ provides an acceptable separation. (4) Single motifs only show *C*_*t*_^*b*^ and *C*_*d*_^*b*^ which both provide moderate separations (Figure 2(d)).

In terms of *C*_*t*_^*w*^ and *C*_*d*_^*w*^, the separability of distributions between known interactions and unapproved DTPs denotes how their distribution meets the popular assumption that similar targets/drugs tend to interact with similar drugs/targets. Our results show that both *C*_*t*_^*w*^ and *C*_*d*_^*w*^ can follow the assumption well and the former is better than the latter.

On the other hand, both *C*_*t*_^*b*^ and *C*_*d*_^*b*^ cannot provide a good separability between known interactions and unapproved pairs. However, they follow our observation that similar drugs, in part, do not tend to target dissimilar proteins. More importantly, in the case of meeting our observation, *C*_*t*_^*b*^ and *C*_*d*_^*b*^ may help in prediction when they are combined with *C*_*t*_^*w*^ and *C*_*d*_^*w*^ together.

**Figure 3:**
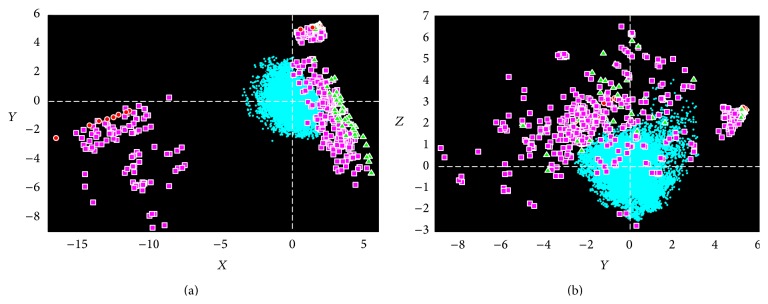
Drug-target pair space. Unapproved DTPs are marked by cyan crosses. Approved DTPs of drug-centered, target-centered, single, and multiple motifs are marked by red circles, green triangles, yellow diamonds, and purple squares, respectively. *X*, *Y*, and *Z* denote the first three principal components, respectively.

Therefore, integrating all four scores together by combination, such as principal component analysis (PCA), can hopefully generate a better separation because known DTIs and unapproved DTPs can be classified in individual dimensions. After performing PCA on these four scores, we showed a space of drug-target pairs on the first three principal components (in Figure 3). In the space, the greatly significant separation between known interactions and unapproved drug-target pairs is observed.

## 3. Result and Discussion

In this section, we shall first demonstrate the effectiveness of our topological similarity metric and our adaptive combination of similarities, compare our approach with other popular methods, including NBI [7] and BLM [8] and its extensions BLM-GIP [9] and BLM-NII [10], build a drug-target interaction space by PCA to elucidate the relationship between known DTIs and unapproved DTPs afterwards, and finally utilize the space to predict the potential interactions for DTPs.

By applying PCA on feature vectors of all drug-target pairs, we used the distances of both known interactions and unapproved pairs to the origin as the confidence scores for both validating the performance of our approach and predicting potential drug-target interactions (more details in Section 3.3). Besides, the popular measurements including area under the curve (AUC) and area under the precision-recall curve (AUPR) [17] were used to assess the computational effectiveness of approaches.

### 3.1. The Effectiveness of New Similarity and New Combination

To illustrate why our approach achieved better results, we first compared GIP similarity and our MI similarity under BLM framework and our approach, respectively. Using the topological similarities only, we selected the sparsest DTI network (NR dataset) from the work [8] to perform the comparison (Table 2). The results demonstrate that our new topological similarity is better than GIP similarity.

Then, we also applied linearly weighted combination to integrate MI with chemical structure similarity/sequence similarity in our approach, respectively. In terms of the values of AUC and AUPR, the linear combination achieved 0.977 and 0.826 while the adaptive combination achieved 0.982 and 0.949. Again, our adaptive combination is better than the linear combination.

### 3.2. Comparison with Other Methods

To validate the effectiveness of our approach, we made a comparison with other approaches [7–10] which adopted the same datasets [13] (see also Section 2.1), the same testing strategy (leave-one-out cross validation, LOOCV), and the same assessment (AUC and AUPR) [17]. First, we run predictions with only chemical similarities of drugs and sequence similarities of targets and compared the results with those of BLM and BLM-GIP. Then after integrating topological similarities, our approach compared with NBI [7], BLM-GIP, and BLM-NII. All results on four datasets are listed in Table 3. In terms of AUC, our approach outperforms on all datasets. In terms of AUPR, our approach has about 7%~10% increase on EN, GPCR, and NR, though it shows ~5% decrease on IC when compared with BLM-NII. Totally, the proposed approach has better predicting performance.

Moreover, our approach has other advantages. First, our approach holds a sufficient number of positive samples (all known DTIs) even if the number of negative samples is large, while BLM may suffer from biased classifier models since each of its local models is trained by few positive samples (even 0 or 1 sample sometimes). Then, our approach only needs to train only one classifier whereas BLM and its extensions need to build many classifiers accounting for all targets and all drugs. Last but most importantly, with the representation of feature vector, we are able to put all drug-target pairs, including the pairs between new drugs and new targets, into the same space regardless of whether the drug and the target in the concerned pair are in the same subnetwork or not. Consequently, our approach is generally superior to other former approaches.

### 3.3. Drug-Target Pair Space and Its Application to Find Potential Interactions

After performing PCA on feature vectors, we represented all DTPs as points shown by their first three principle components (denoted by *X*, *Y*, and *Z* in Figure 3, resp.). Approved DTPs (DTIs) and unapproved DTPs show two separating groups. The unapproved DTPs (cyan crosses) gather around the origin in a sphere-like shape while the known DTIs were apart from them. Particularly in Figure 3(a), three clusters of interaction motifs are found. The cluster in left contains drug-centered motif (red circles) and multiple motifs (purple squares), and the lower cluster in right comprises target-centered motifs and multiple motifs and the upper cluster in right is composed of all four types of motifs.

The significant distribution of DTPs in the space allows us to visually investigate the relationship between known DTIs and unapproved DTPs. Therefore, after calculating the distances of all pairs to the origin, we are not only able to build classifiers by training a specific threshold of the distances when testing the performance of our proposed method (refer to Sections 3.1 and 3.2) but are also able to adopt them as the confidence scores of being potential interactions when predicting potential interactions for unapproved DTPs.

According to the distribution in DTP space, the farther the pair is from the origin, the more possible it is to be a potential interaction. Thus, we only focused on the unapproved drug-target pairs remarkably far away from the origin. In order to validate them, we selected the top five out of them as the interaction candidates in terms of their distance to the origin for each dataset and checked them in popular drug/compound databases, ChEMBL (C), DrugBank (D), and KEGG (K). Since ChEMBL provides the predicted interactions (not approved yet), we only selected the most confident interactions with the score of 1 under the cut-off of 1 *μ*M [5]. Comparing with ChEMBL, DrugBank, and KEGG, we showed our consistent predictions of the potential interactions of unapproved drug-target pairs for the adopted datasets in Table 4.

## 4. Conclusions

In this paper, we have addressed crucial issues in predicting drug-target interactions, which have not yet been solved well by former methods. These issues include the powerless inference in the case of isolated subnetworks, the biased classifiers derived from few positive samples, the need of training a number of classifiers, and the unavailable relationship between known DTIs and unapproved DTPs.

By characterizing each drug-target pair as a feature vector of within-scores and between-scores, our approach has the following advantages: (1) all types of drug-target pairs are treated in a same form, regardless of the available path between drugs and targets; (2) enough positive samples are able to reduce the bias of training model and only one classifier needs to be trained; (3) more importantly, the relationship between known DTIs and unapproved DTPs can be investigated in the same visualized space.

In addition, to capture similarity better, we have introduced a complete metric of topological similarity of drugs/targets by considering both the targets/drugs shared by two drugs/targets and the targets/drugs interacting with none of them. We also have proposed an adaptive combination rule, instead of the former linear combination between topological similarity and chemical/sequence similarity, by considering that the drug/target nodes' degrees follow the power-law distribution.

Finally, the effectiveness of our approach is demonstrated by comparing with existing popular methods under the cross validation and predicting potential interactions for DTPs under the validation in existing databases.

## Supplementary Material

Table S1 lists the counts of four types of motifs, including multiple, drug-centered, target-centered, and single interacting motifs, in four adopted datasets. Fig S1, S2, S3 and S4 show the distributions of the proposed features of four different motifs by histograms respectively. The distributions of known drug-target interactions and unapproved drug-target pairs are rendered in different colors.

## Figures and Tables

**Table 1:** Four datasets used in this work.

Dataset name	#Drugs	#Targets	#Interactions	Proportion of unreachable paths between drugs	Proportion of unreachable paths between targets
EN	445	664	2926	0.479	0.479
IC	210	204	1476	0.019	0.029
GPCR	223	95	635	0.345	0.593
NR	54	26	90	0.615	0.778

# denotes the number of drugs, targets, or drug-target interactions in dataset.

**Table 2:** Comparison between topological similarities.

	GIP (AUC/AUPR)	MI (AUC/AUPR)
BLM	**0.662/0.321**	**0.762/0.434**
Ours	**0.918/0.757**	**0.949/0.786**

**Table 3:** Comparison with other three methods under LOOCV.

	BLM^*^	BLM-GIP^*^	Our^*^	NBI#	BLM-GIP#	BLM-NII#	Our#
EN	0.976/0.833	0.966/0.845	**0.985/0.849**	0.975	0.978/0.915	0.988/0.929	**0.999/0.998**
IC	0.973/0.781	0.971/0.807	**0.977/0.820**	0.976	0.984/0.943	0.990/**0.950**	**0.997/**0.897
GPCR	0.955/0.667	0.947/0.660	**0.975/0.772**	0.946	0.954/0.790	0.984/0.865	**0.998/0.971**
NR	0.881/0.612	0.864/0.547	**0.946/0.774**	0.838	0.922/0.684	0.981/0.866	**0.982/0.949**

^*^Using chemical similarity for drugs and sequence similarity for targets only.

#Combining topological similarities (MI) with chemical similarity and sequence similarity, respectively. NBI only provides AUC values and run tests under 5-fold cross validation (5CV) which is statistically same as LOOCV when the number of samples is enough.

**Table 4:** The top five predicted interactions of nuclear receptor.

Rank	En		IC		GPCR		NR	
	Validation	Pair	Validation	Pair	Validation	Pair	Validation	Pair
1	D	D05458 hsa:4128	D, K	D00438 hsa779	C	D03966 hsa2914	C, K	D00348 hsa5915

2	D	D00947 hsa:4129	—	D00619 hsa3749	—	D03966 hsa2917	C, K	D00348 hsa5916

3	—	D00039 hsa:587	—	D00816 hsa3781	—	D01346 hsa2916	—	D01132 hsa6097

4	—	D00437 hsa:1585	D	D00619 hsa776	K	D00442 hsa6755	C	D00348 hsa6256

5	—	D03365 hsa:1548	—	D00619 hsa3736	—	D00049 hsa8843	C	D00348 hsa6257

C, D, and K label the validated interactions in ChEMBL, DrugBank, and KEGG, respectively.
