# Supplementary material for: Predicting Drug-Target Interactions via Within-Score and Between-Score
Source: Biomed Res Int. 2015 Oct 12;2015:350983. doi: 10.1155/2015/350983 (PMC4620248; doi:10.1155/2015/350983)
Supplement: Supplementary file 1 — Table S1 lists the counts of four types of motifs, including multiple, drug-centered, target-centered, and single interacting motifs, in four adopted datasets. Fig S1, S2, S3 and S4 show the distributions of the proposed features of four different motifs by histograms respectively. The distributions of known drug-target interactions and unapproved drug-target pairs are rendered in different colors. [file 350983.f1.docx]

**Supplementary**

| Table S1. The statistics of interaction motifs | | | | |
| --- | --- | --- | --- | --- |
| Datasets(interactions) | Multiple | Drug-centered | Target-centered | Single |
| EN (2926) | 2475 | 274 | 163 | 14 |
| IC (1476) | 1373 | 22 | 80 | 1 |
| GPCR (635) | 503 | 26 | 98 | 8 |
| NR (90) | 46 | 5 | 36 | 3 |

| 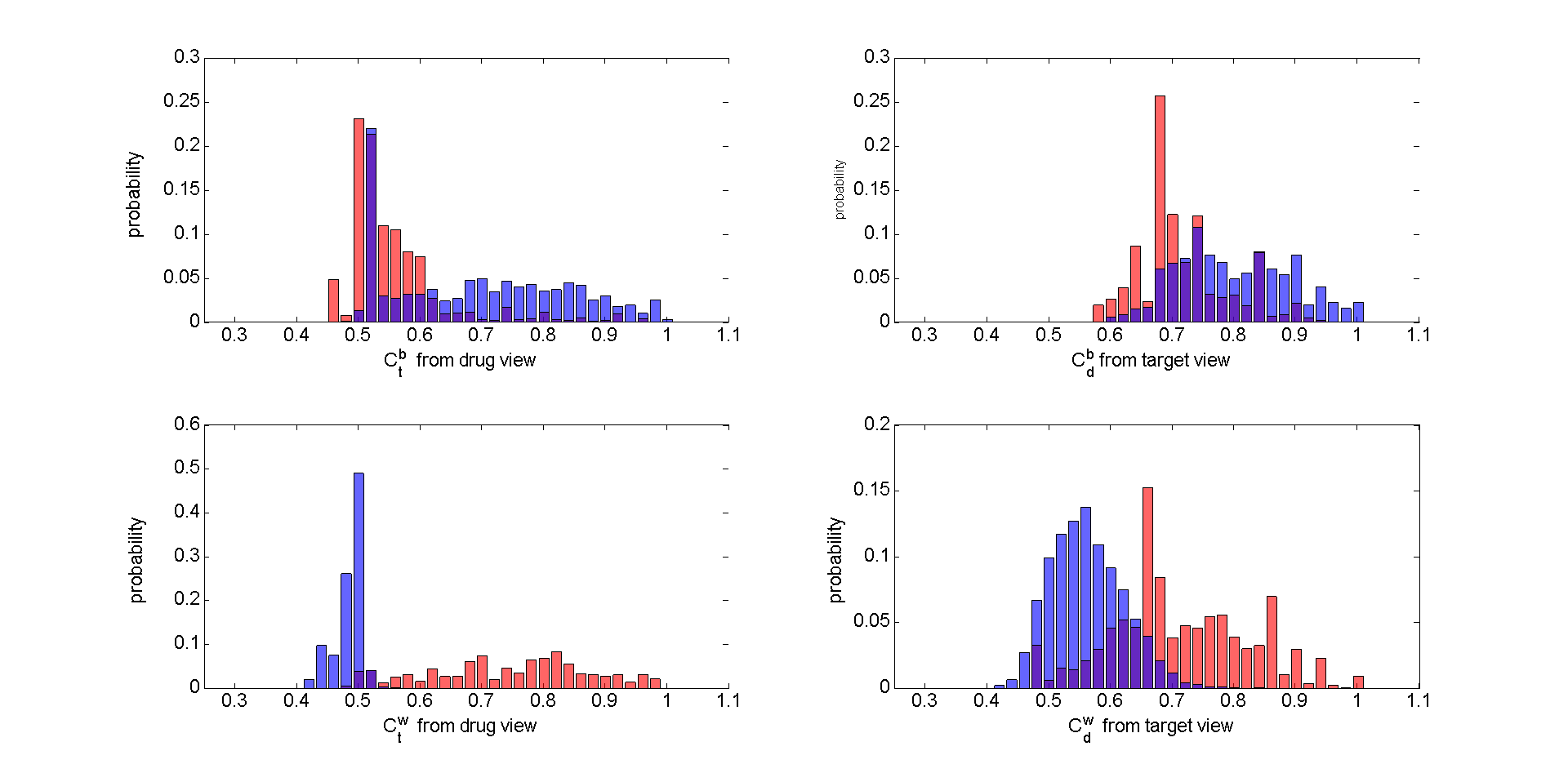 |
| --- |
| (a) EN |
| 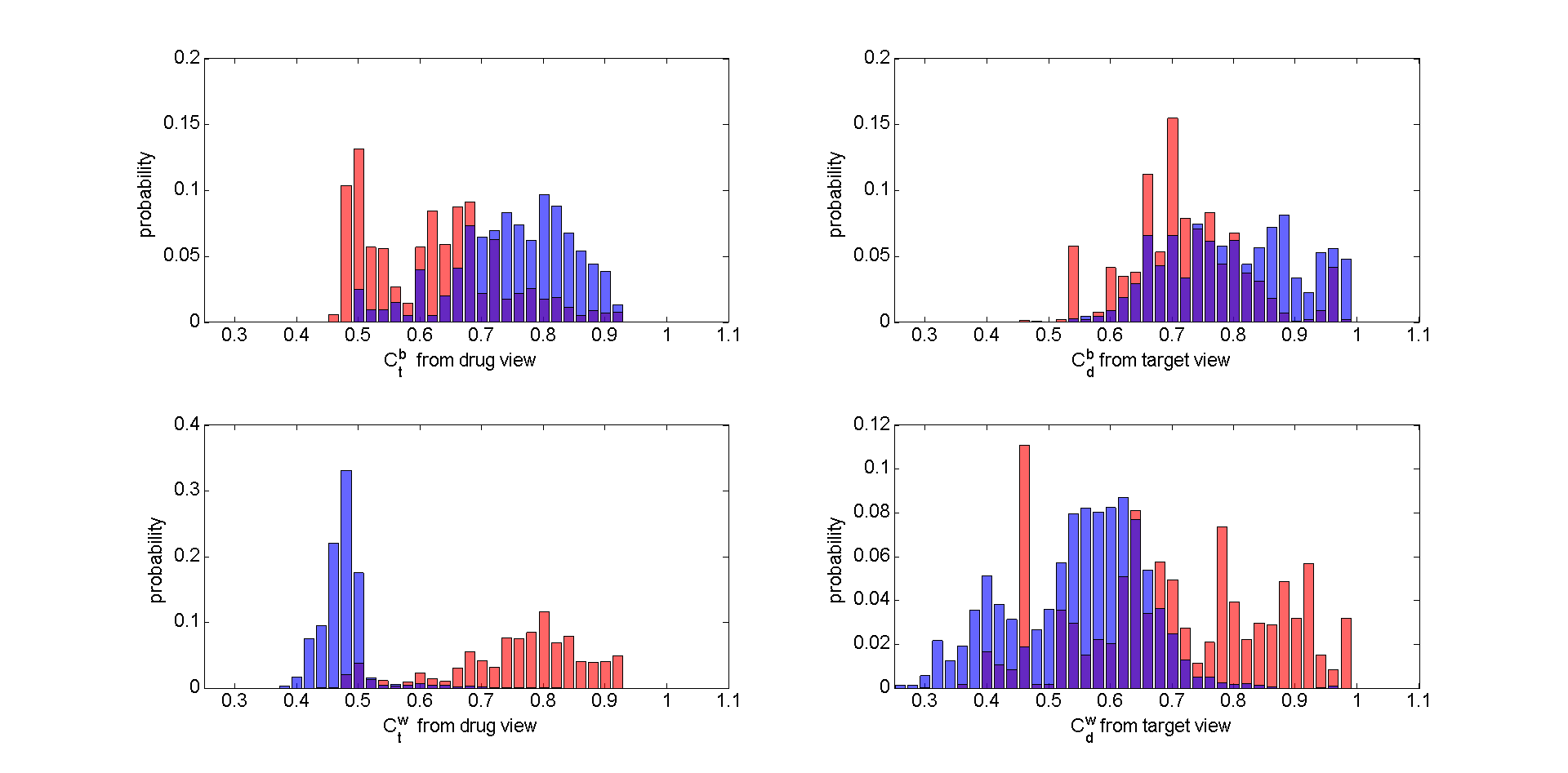 |
| (b) IC |
| 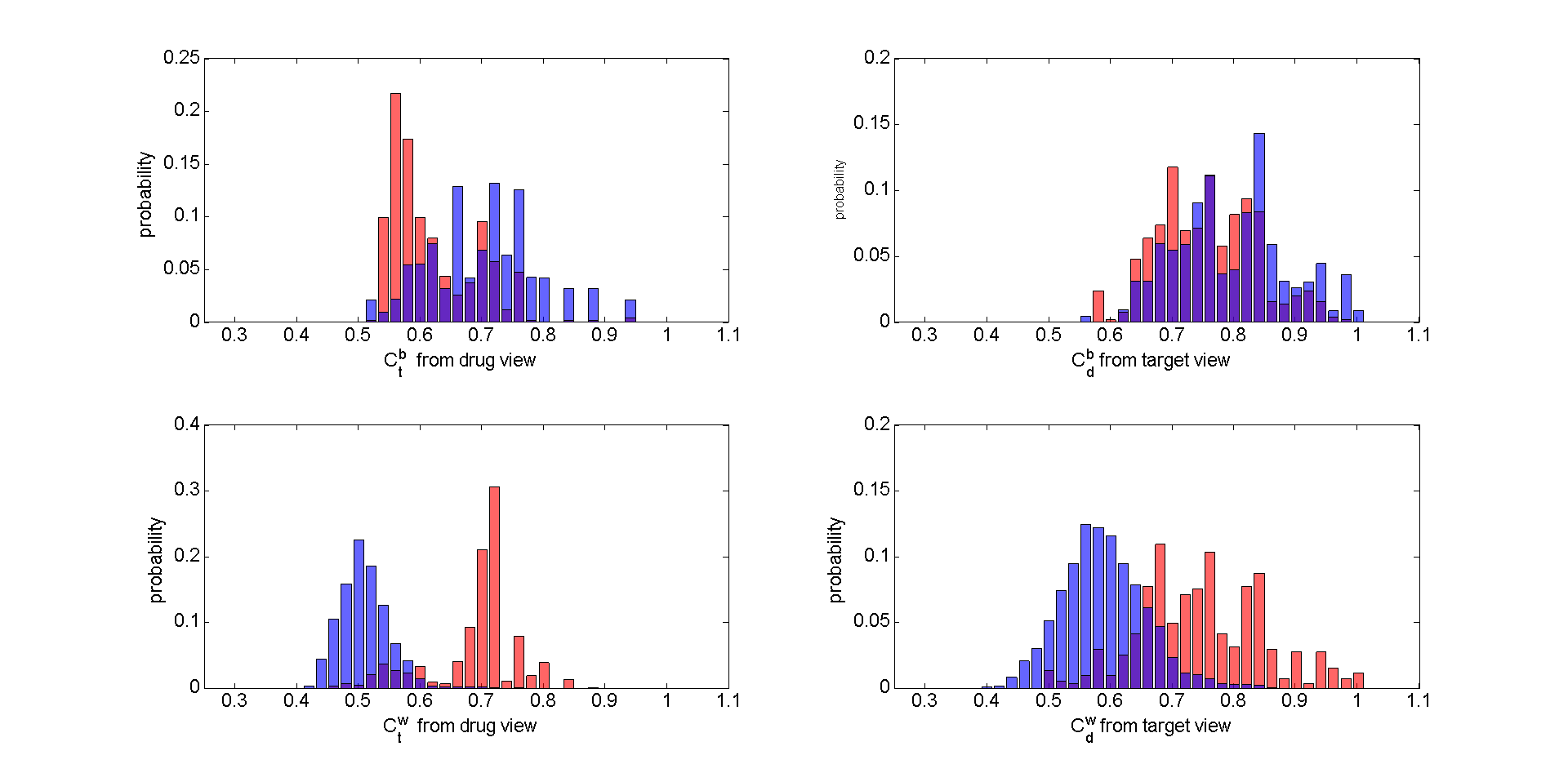 |
| (c) GPCR |
| 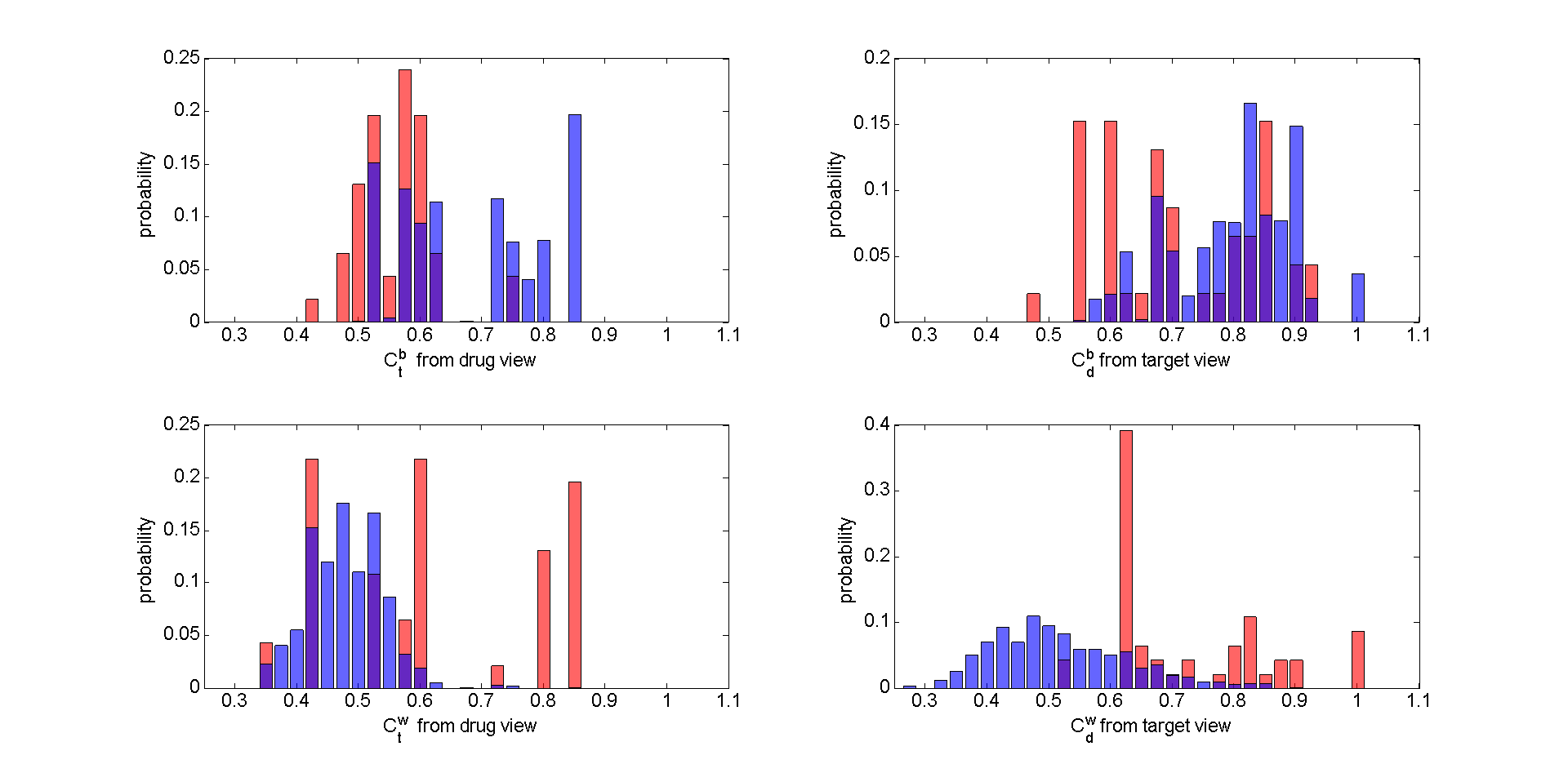 |
| (d) NR |
| Fig. S1. The distributions of Multiple motifs in four datasets. The distributions of known DTIs and unapproved DTPs are rendered in red and blue and their overlapping parts are rendered in the mix of red and blue. |

| 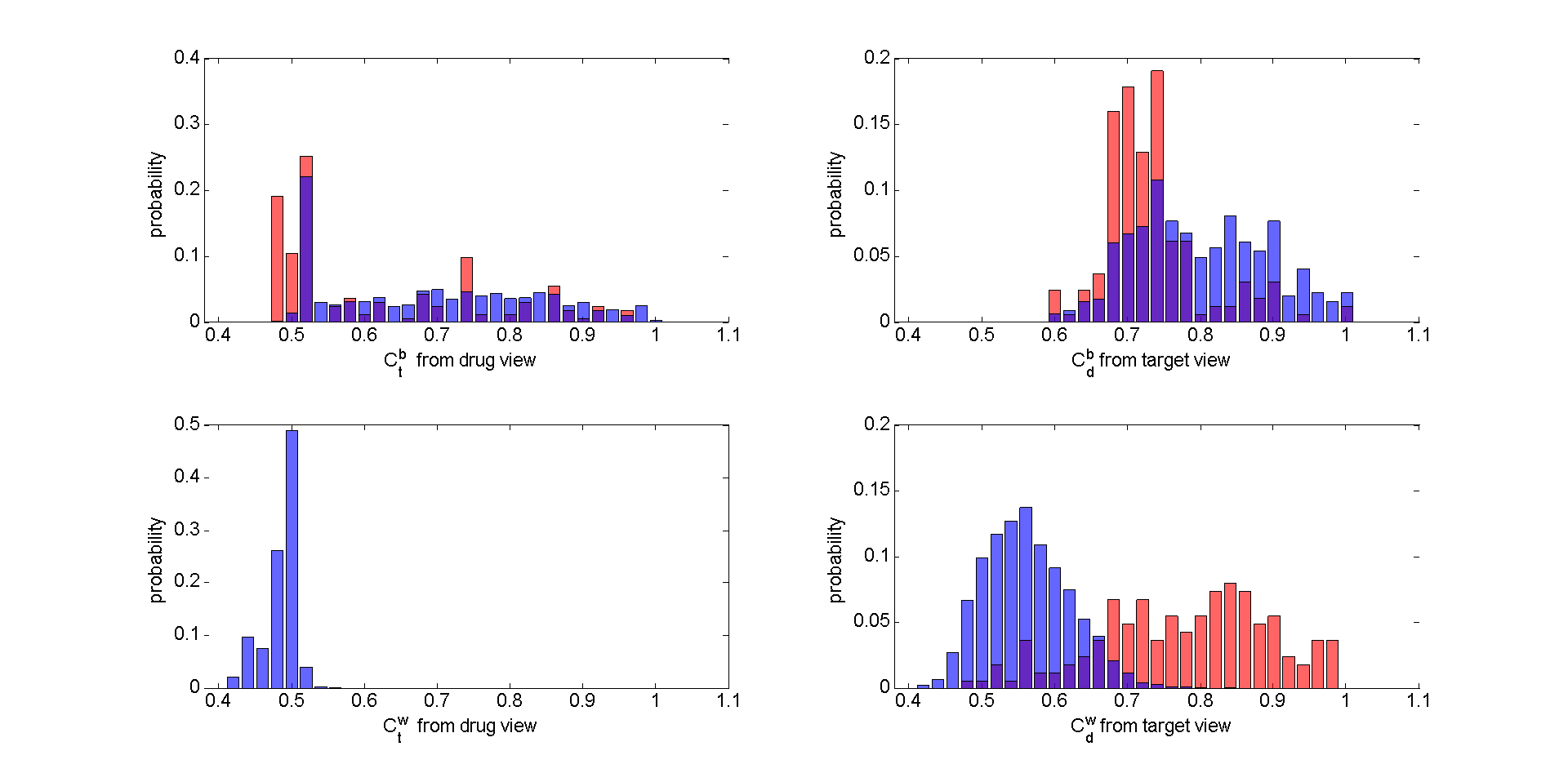 |
| --- |
| (a) EN |
| 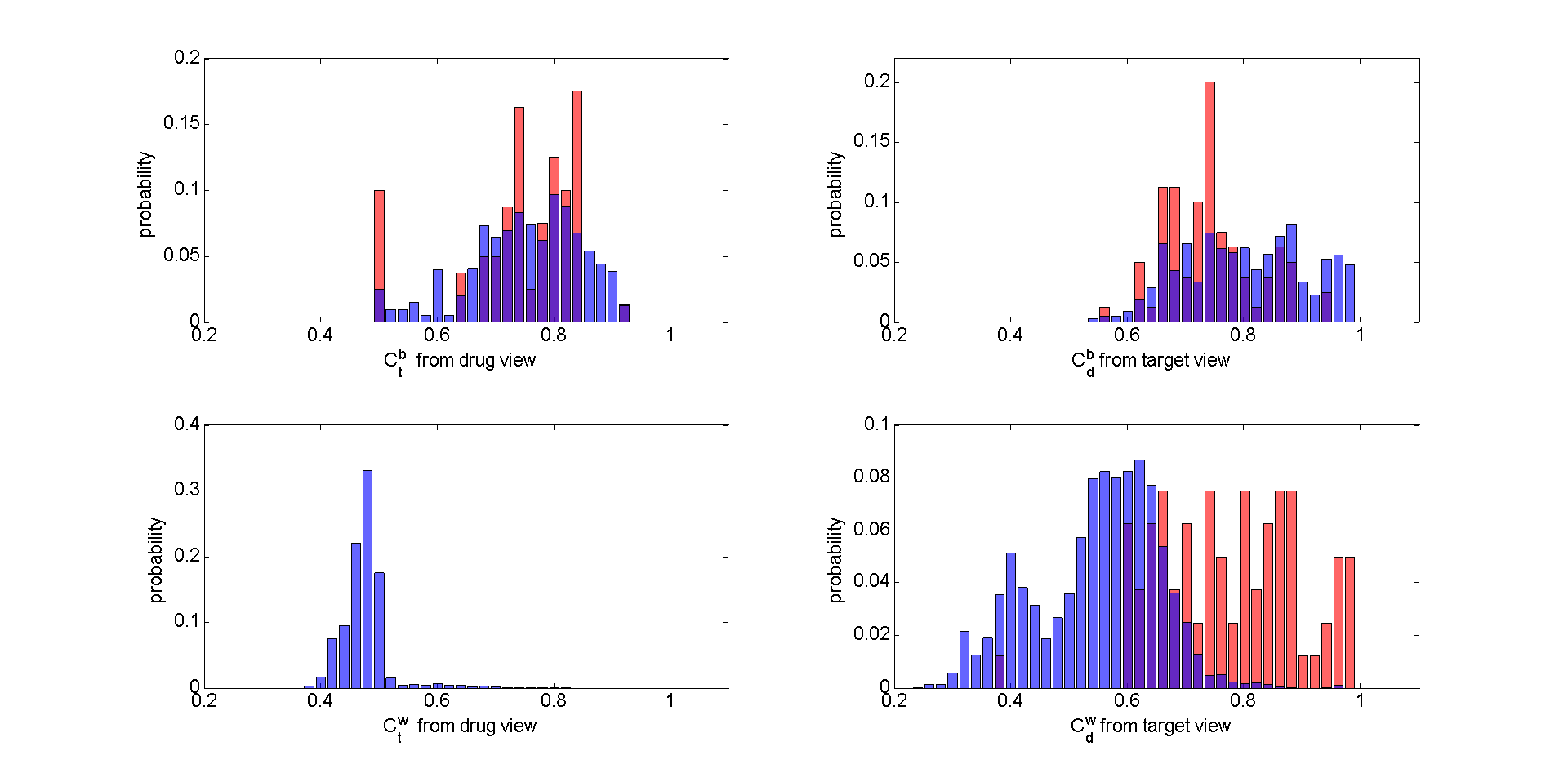 |
| (b) IC |
| 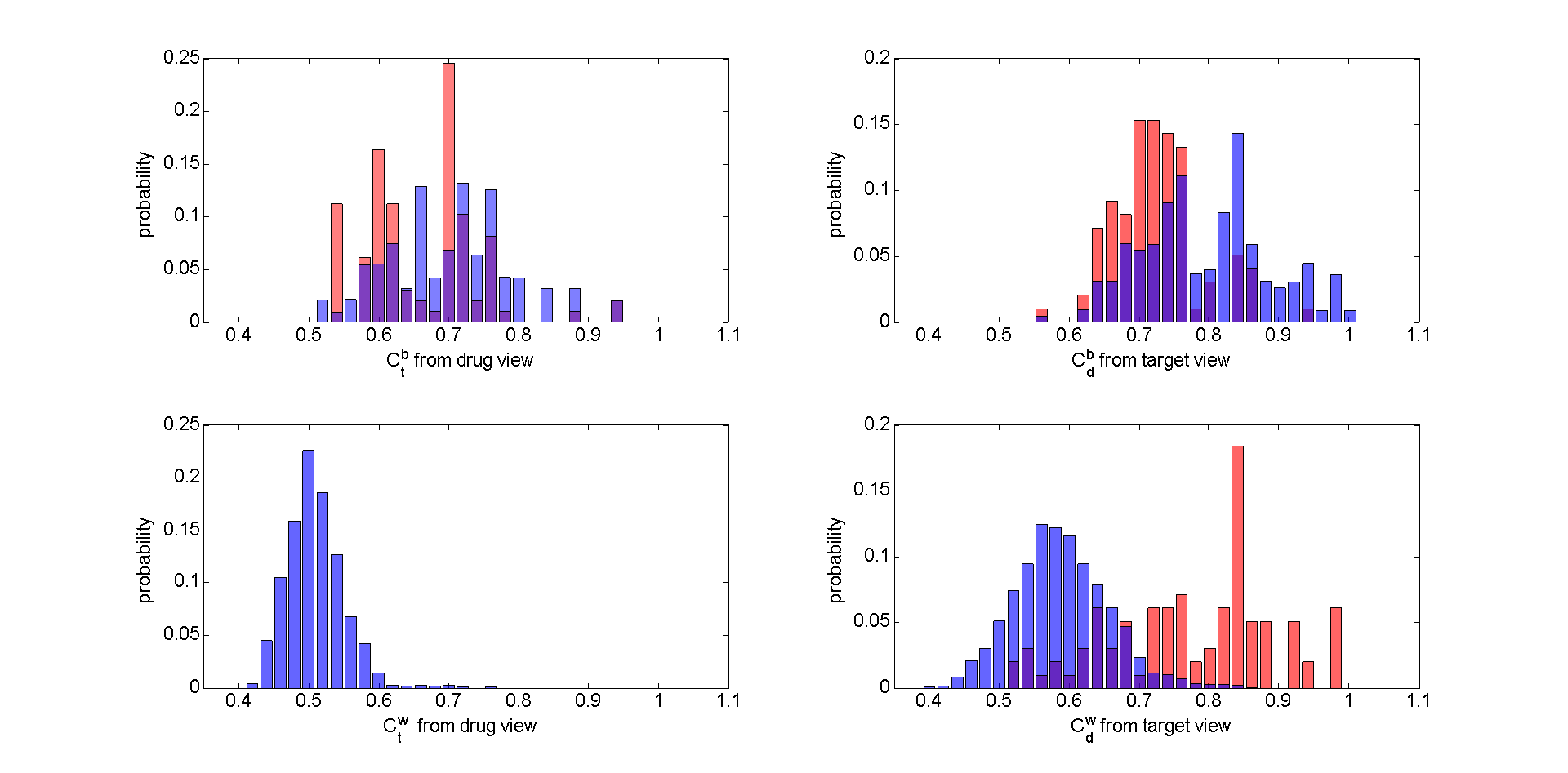 |
| (c) GPCR |
| 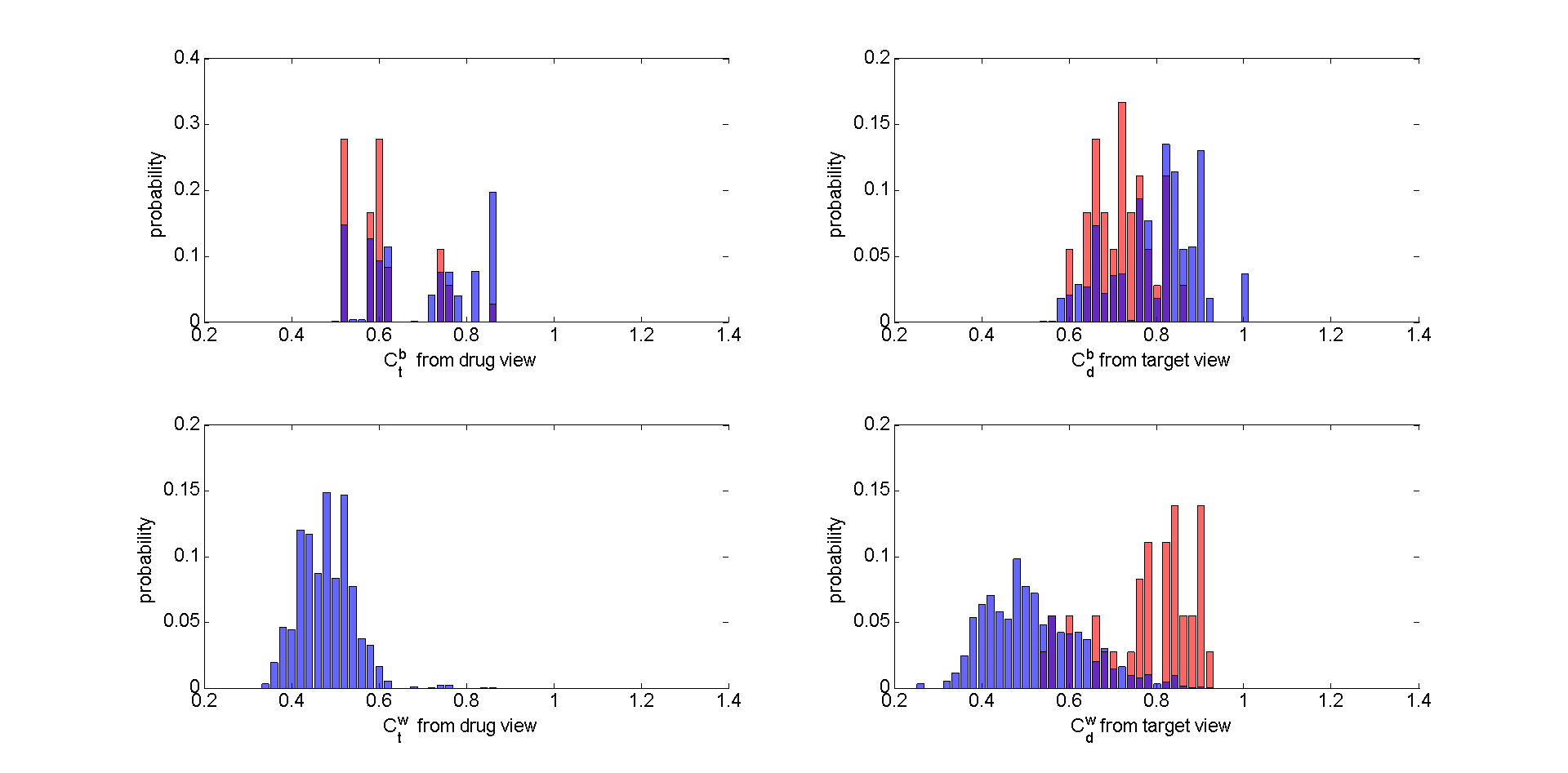 |
| (d) NR |
| Fig. S2. The distributions of Target-centered motifs in four datasets. The distributions of known DTIs and unapproved DTPs are rendered in red and blue and their overlapping parts are rendered in the mix of red and blue. |

| 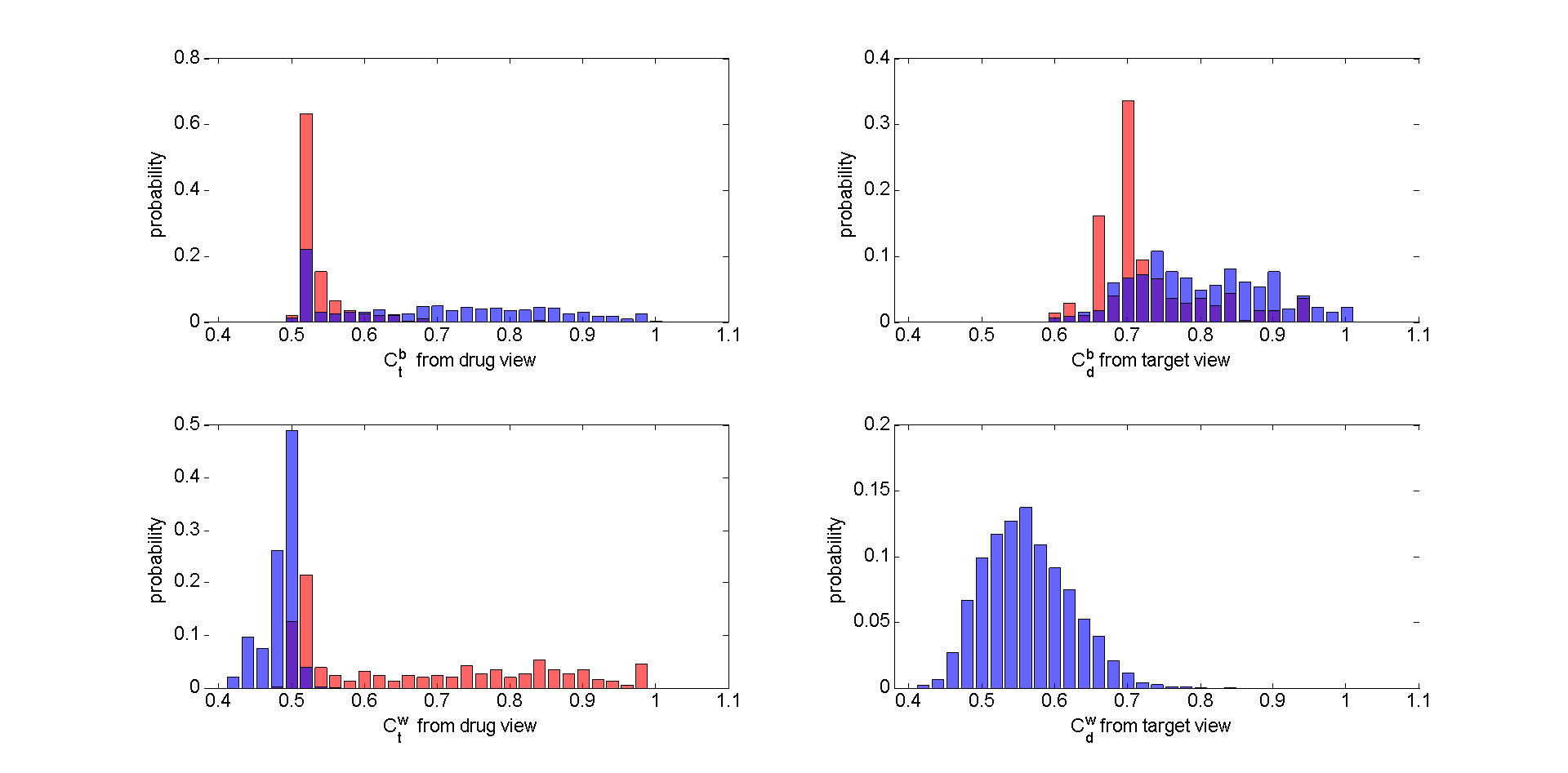 |
| --- |
| (a) EN |
| 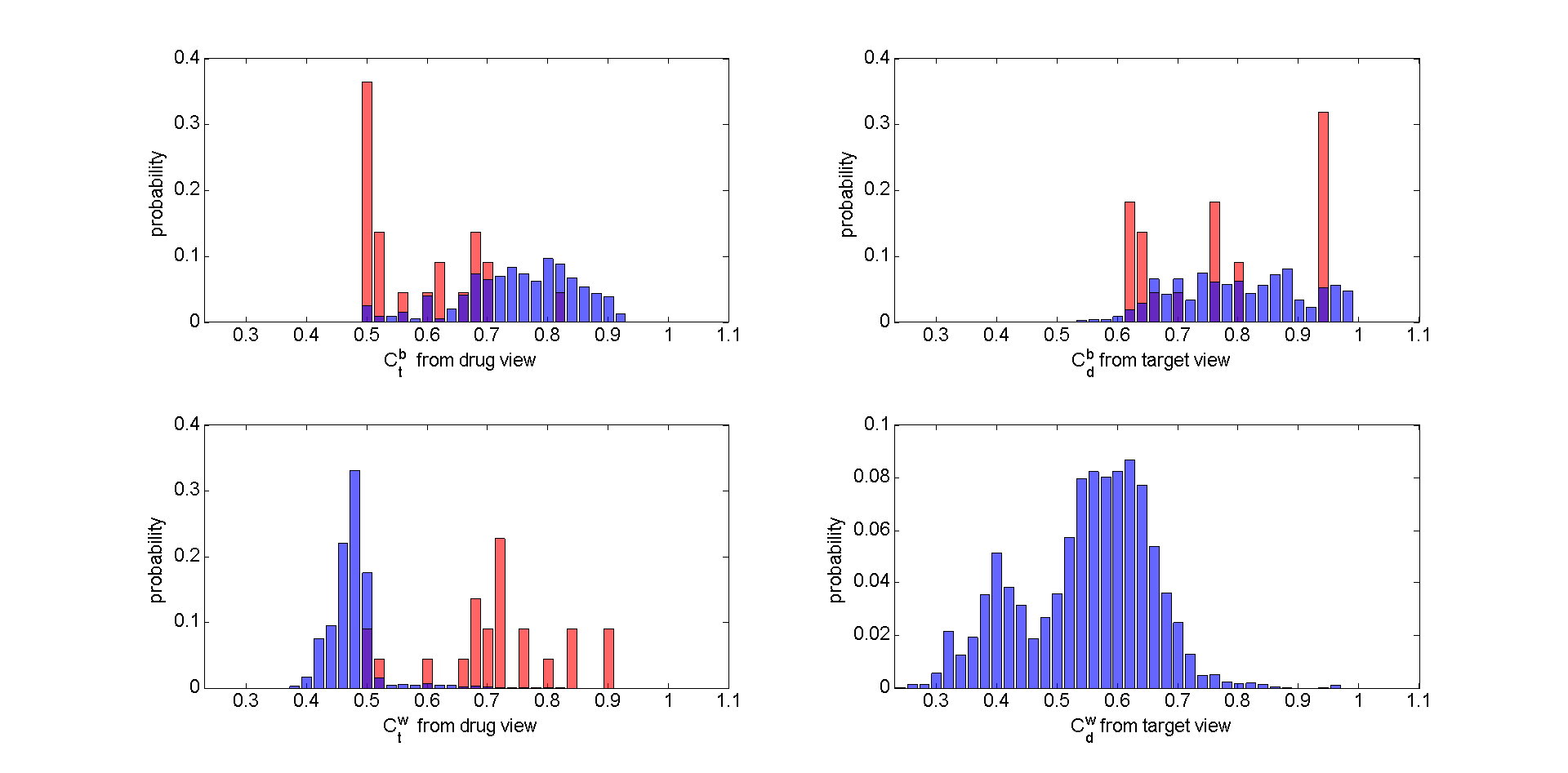 |
| (b) IC |
| 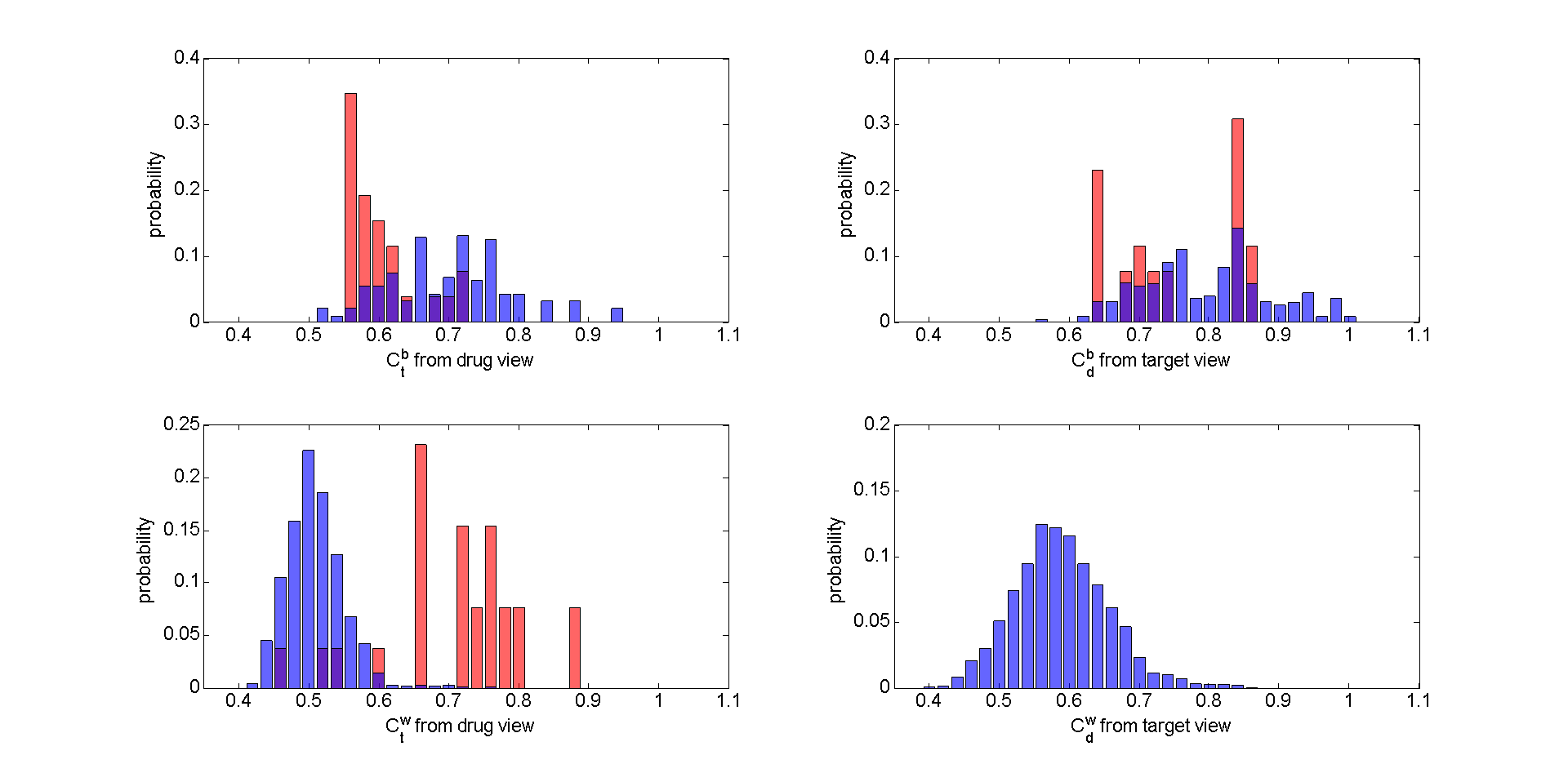 |
| (c) GPCR |
| 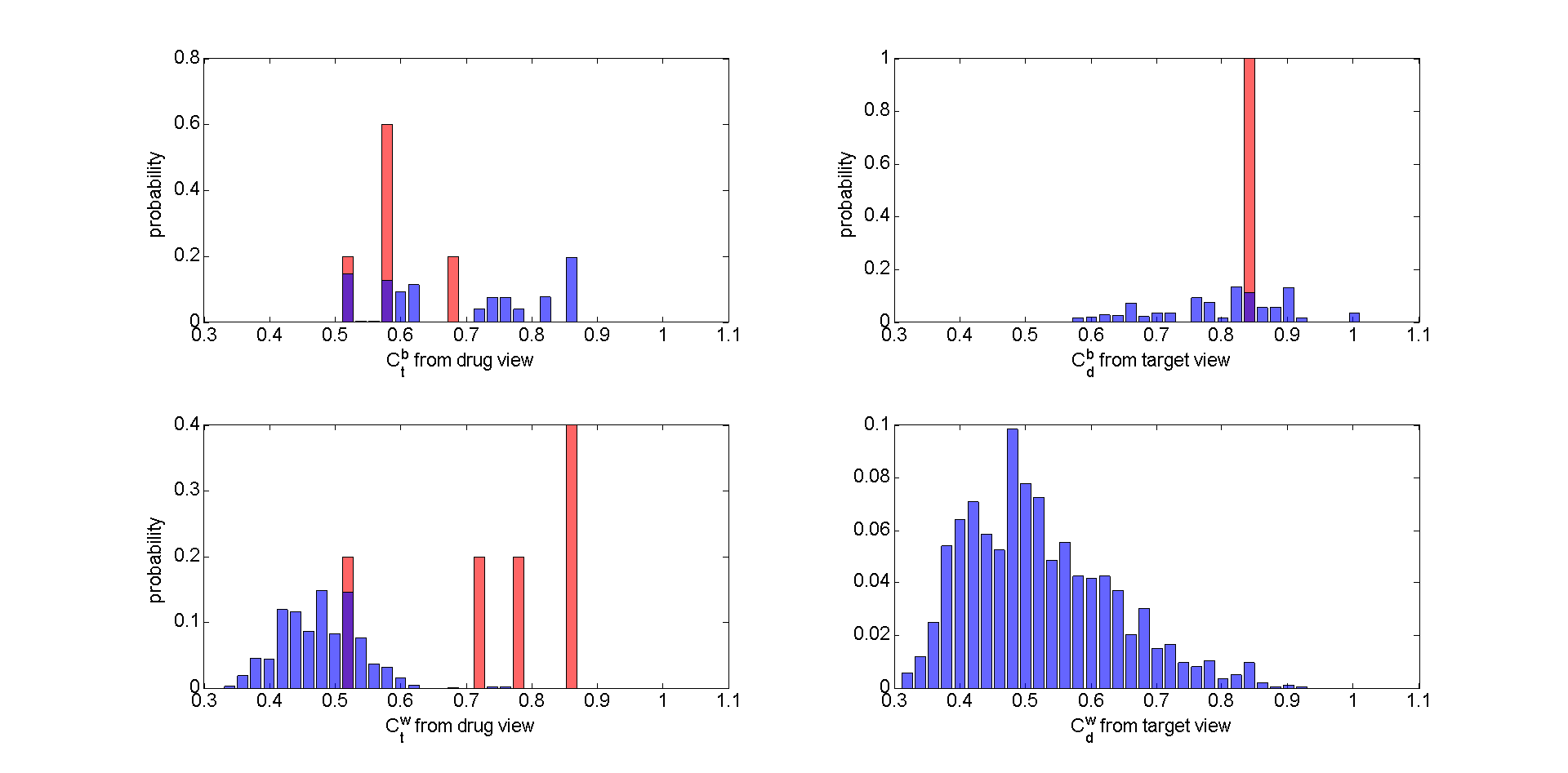  0.2:0.01:1 |
| (d) NR |
| Fig. S3. The distributions of Drug-centered motifs in four datasets. The distributions of known DTIs and unapproved DTPs are rendered in red and blue and their overlapping parts are rendered in the mix of red and blue. |

| 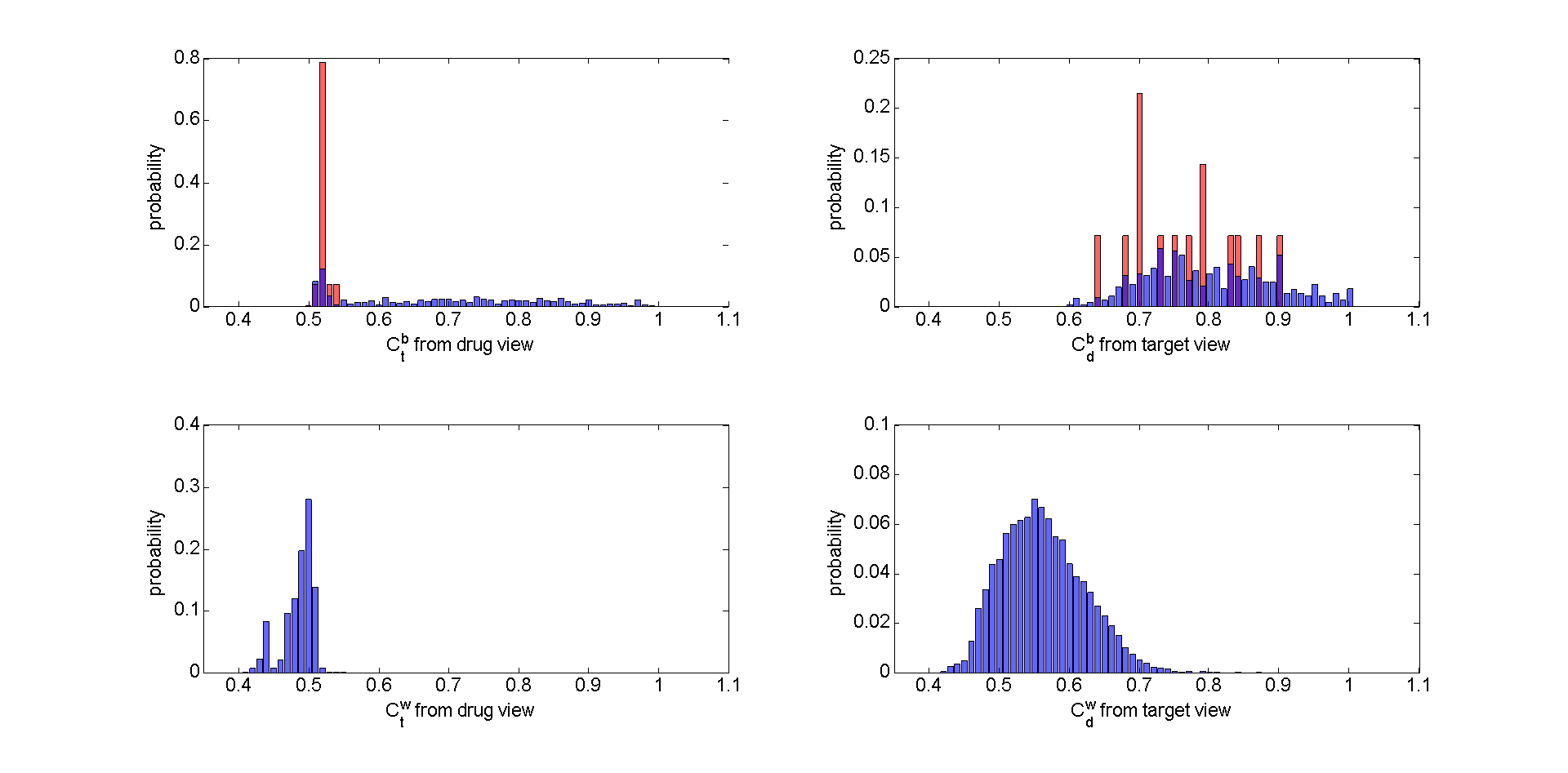 |
| --- |
| (a) EN |
| 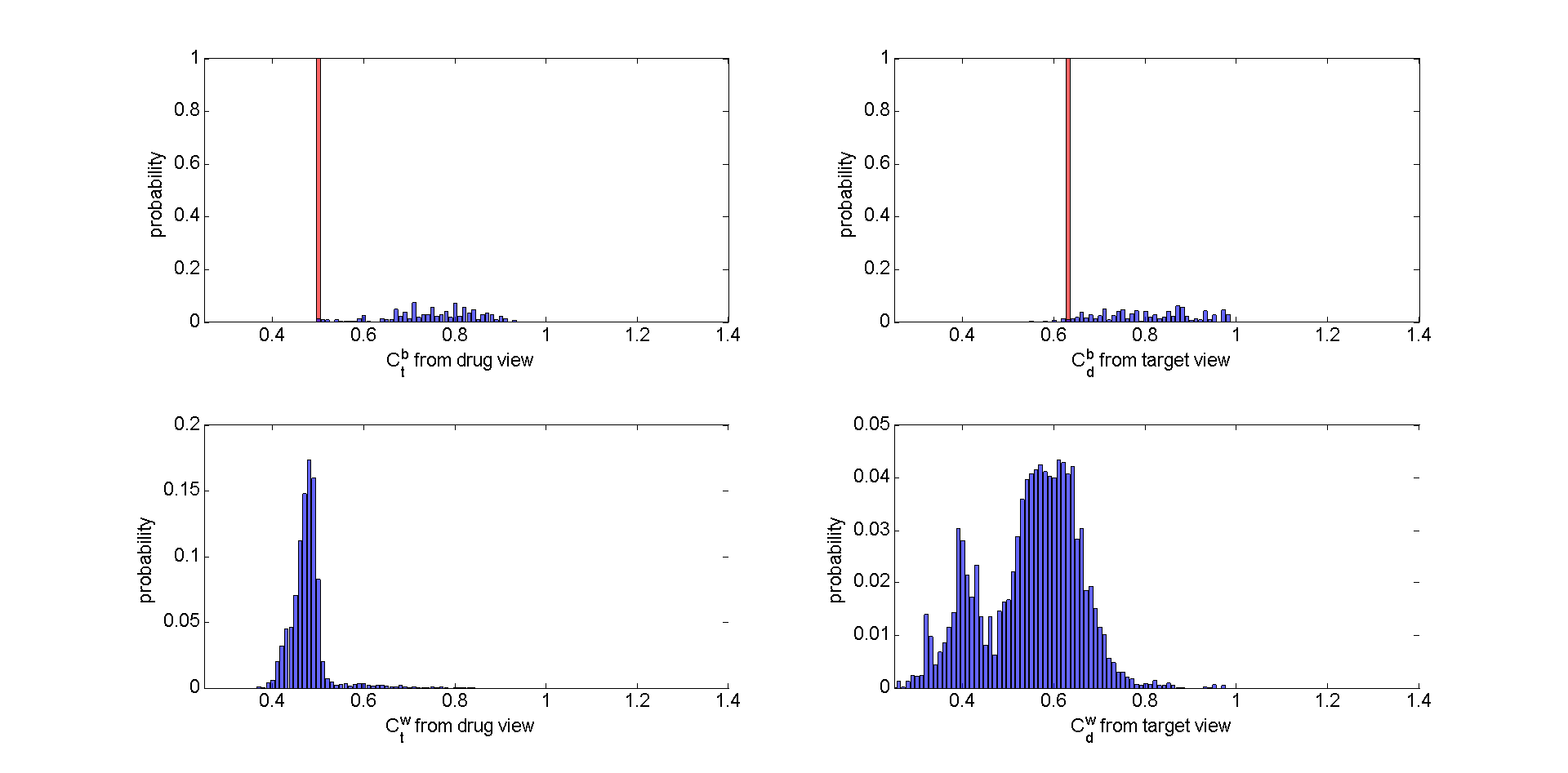 |
| (b) IC |
| 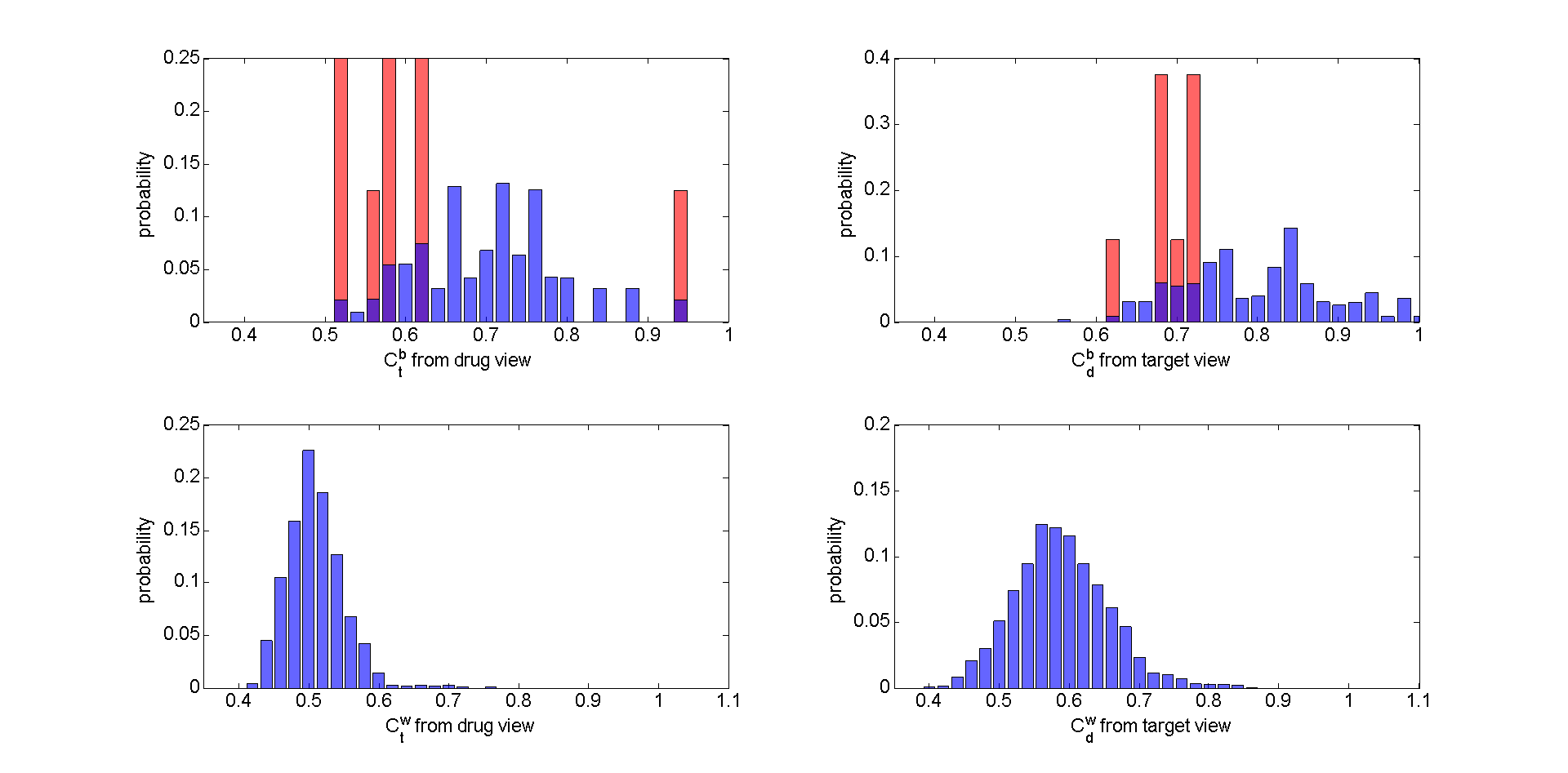 |
| (c) GPCR |
| 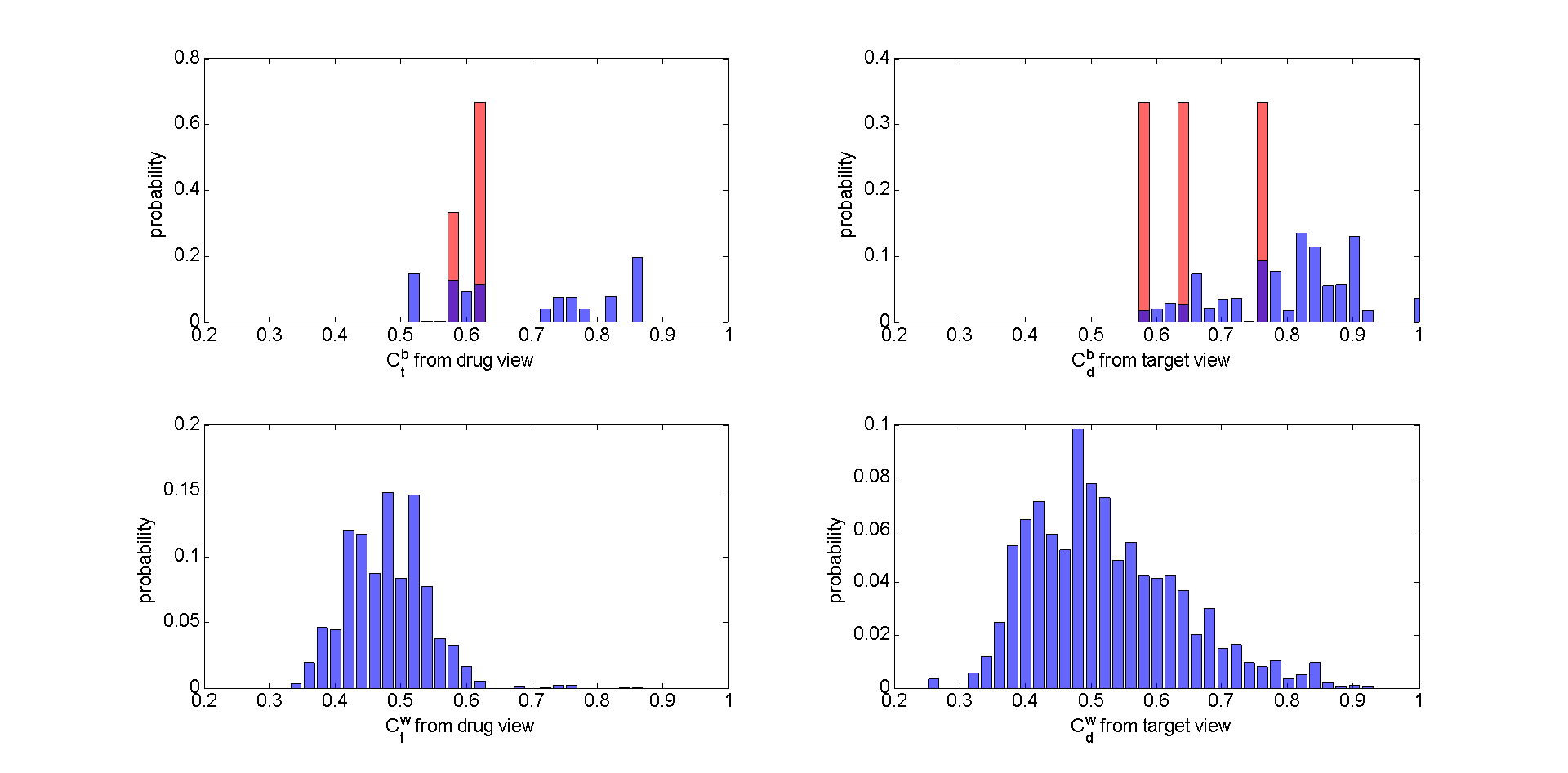 |
| (d) NR |
| Fig. S4. The distributions of Single motifs in four datasets. The distributions of known DTIs and unapproved DTPs are rendered in red and blue and their overlapping parts are rendered in the mix of red and blue. |
